# Extracorporeal membrane oxygenation in adult patients with hematologic malignancies and severe acute respiratory failure

**DOI:** 10.1186/cc13701

**Published:** 2014-01-20

**Authors:** Philipp Wohlfarth, Roman Ullrich, Thomas Staudinger, Andja Bojic, Oliver Robak, Alexander Hermann, Barbara Lubsczyk, Nina Worel, Valentin Fuhrmann, Maria Schoder, Martin Funovics, Werner Rabitsch, Paul Knoebl, Klaus Laczika, Gottfried J Locker, Wolfgang R Sperr, Peter Schellongowski, Arbeitsgruppe für hämato-onkologische Intensivmedizin der Österreichischen Gesellschaft für Internistische und Allgemeine Intensivmedizin und Notfallmedizin (ÖGIAIN)

**Affiliations:** Department of Medicine I, Intensive Care Unit 13i2, Comprehensive Cancer Center, Medical University of Vienna, Waehringer Guertel 18-20, A-1090 Vienna, Austria; Department of Anaesthesia, Intensive Care Unit 13c2, Medical University of Vienna, Waehringer Guertel 18-20, A-1090 Vienna, Austria; Department of Bloodgroup Serology and Transfusion Medicine, Medical University of Vienna, Waehringer Guertel 18-20, A-1090 Vienna, Austria; Department of Medicine III, Intensive Care Unit 13h1, Medical University of Vienna, Waehringer Guertel 18-20, A-1090 Vienna, Austria; Department of Biomedical Imaging and Image-guided Therapy, Division of Cardiovascular and Interventional Radiology, Medical University of Vienna, Waehringer Guertel 18-20, A-1090 Vienna, Austria

## Abstract

**Introduction:**

Acute respiratory failure (ARF) is the main reason for intensive care unit (ICU) admissions in patients with hematologic malignancies (HMs). We report the first series of adult patients with ARF and HMs treated with extracorporeal membrane oxygenation (ECMO).

**Methods:**

This is a retrospective cohort study of 14 patients with HMs (aggressive non-Hodgkin lymphoma (NHL) *n* = 5; highly aggressive NHL, that is acute lymphoblastic leukemia or Burkitt lymphoma, *n* = 5; Hodgkin lymphoma, *n* = 2; acute myeloid leukemia, *n* = 1; multiple myeloma, *n* = 1) receiving ECMO support because of ARF (all data as medians and interquartile ranges; age, 32 years (22 to 51 years); simplified acute physiology score II (SAPS II): 51 (42 to 65)). Etiology of ARF was pneumonia (*n* = 10), thoracic manifestation of NHL (*n* = 2), sepsis of nonpulmonary origin (*n* = 1), and transfusion-related acute lung injury (*n* = 1). Diagnosis of HM was established during ECMO in four patients, and five first received (immuno-) chemotherapy on ECMO.

**Results:**

Before ECMO, the PaO_2_/FiO_2_ ratio was 60 (53 to 65), (3.3 to 3.7). Three patients received venoarterial ECMO because of acute circulatory failure in addition to ARF; all other patients received venovenous ECMO. All patients needed vasopressors, and five needed hemofiltration. Thrombocytopenia occurred in all patients (lowest platelet count was 20 (11 to 21) G/L). Five major bleeding events were noted. ECMO duration was 8.5 (4 to 16) days. ICU and hospital survival was 50%. All survivors were alive at follow-up (36 (10 to 58) months); five patients were in complete remission, one in partial remission, and one had relapsed.

**Conclusions:**

ECMO therapy is feasible in selected patients with HMs and ARF and can be associated with long-term disease-free survival.

## Introduction

The acute respiratory failure (ARF) represents the predominant reason for medical intensive care unit (ICU) admissions in patients with hematologic malignancies (HMs) [1–5]. Its occurrence has a strong prognostic impact, especially if mechanical ventilation becomes necessary. Although the outcome of those affected was described to be dismal in the past, survival improved markedly in recent years. This development may be attributed to advances in patient selection [6], general improvements in the management of the acute respiratory distress syndrome (ARDS), as well as specific improvements in managing ARF in patients with HMs. The latter comprises specific diagnostic algorithms in ARF of HM patients [7], as well as the use of noninvasive ventilation early in the course, even though the efficacy of this procedure does not remain undisputed [8–11]. However, mortality rates of patients with HMs and invasive mechanical ventilation due to ARF still exceed 50% [1, 3–5]. Yet, a general reluctance to admit critically ill patients with HMs cannot be justified. Unlimited intensive care has been advocated for selected patients with HMs [12, 13].

The extracorporeal membrane oxygenation (ECMO) depicts one of the ultimate therapies in intensive care and may possibly be beneficial in patients with ARDS in general ICU populations [14, 15]. Some data exist on the use of ECMO in cohorts of children with malignant diseases [16, 17], but the published experience with ECMO in adult patients with HM is limited to two single cases [18, 19]. Because both, patients with HMs and those undergoing ECMO are prone to acquire severe complications, such as bleeding and infection [1, 5, 20–23], performing ECMO in patients with HMs might bear a particularly high risk.

The purpose of this analysis is to report the characteristics and outcomes of patients with HMs and ARF treated with ECMO.

## Materials and methods

We retrospectively studied the clinical courses of all adult patients (18 years or older) with HMs and ARF treated with ECMO at the Medical University of Vienna, General Hospital, between September 2000 and June 2013. This study was conducted in accordance with the amended Declaration of Helsinki. The ethics committee of the Medical University of Vienna approved the protocol and waived the need for informed consent because of the noninterventional retrospective design of the investigation.

The presence of ARDS was defined and graded according to recently introduced criteria [24]. The term baseline refers to the time immediately before the start of ECMO treatment. Thrombocytopenia was defined as platelet count <150 G/L, and leukocytopenia as leukocyte count <4 G/L.

In our center, extracorporeal lung support is usually evaluated in patients presenting with severe and life-threatening hypoxemia while being mechanically ventilated with adequately high positive end-expiratory pressure (PEEP) and a missing response to supportive measures like prone positioning. During ECMO, a maximally achievable protective ventilation setting is desired. Weaning from ECMO is performed according to current Extracorporeal Life Support Organization (ELSO) guidelines [25].

At baseline, we recorded age, gender, characteristics of the hematologic malignancy, including type and stage of the respective malignancy, time of diagnosis, type and timing of previous specific treatments (that is, chemo-, immuno-, or radiotherapy, autologous or allogeneic stem cell transplantation (ASCT)), as well as the intention of cancer treatment (curative versus noncurative). The Charlson Comorbidity Index [26] (CCI) was assessed to account for comorbid conditions. To grade the severity of illness, we calculated the Simplified Acute Physiology Score II (SAPS II) [27, 28] at ICU admission, as well as the Sequential Organ Failure Assessment (SOFA) score [29] at baseline, respectively. We determined the etiology of ARF and recorded arterial blood gas parameters (pH, partial pressures of oxygen (PaO_2_) and carbon dioxide (PaCO_2_), lactate), PaO_2_/FiO_2_ ratio, the Lung Injury Score (LIS) [30], laboratory tests such as white blood cell count, platelet count, hemoglobin, prothrombin time, and fibrinogen, all at baseline, eventually.

As follow-up variable, we recorded type of ECMO (that is, venovenous (VV) versus venoarterial (VA), use of anticoagulants, vasopressors, and blood products, duration of ECMO therapy, length of ICU and hospital stay, bleeding complications (occurrence, location, and severity), other procedure-related complications (trauma or bleeding related to the insertion of cannulas, clotting events), ICU and hospital survival, course of the malignancy (remission status), death date (if applicable), as well as date of the last visit. Bleeding was considered to be major in patients with a decrease in hemoglobin levels >2.0 g/dl or requiring more than 2 units of packed red blood cells due to an obvious bleeding event, surgical interventions, or in cases of intracerebral hemorrhage. In patients with more than one episode of ECMO, baseline parameters and definite outcomes associated with the last respective ECMO episode were analyzed, whereas for calculation of total ECMO days, all episodes were counted.

Continuous data are presented as median and interquartile ranges (25% to 75%), unless otherwise indicated. Dichotomous data are presented as number and percentage. Data were compared between survivors and nonsurvivors with Fisher Exact test for dichotomous variables, and the Mann–Whitney *U* Test for continuous variables, respectively. Differences were considered to be statistically significant when *P* was <0.05.

## Results

We report on 14 consecutive patients with HMs and ARF treated a total number of 17 ECMO episodes. The male/female ratio was 8:6, age 32 years (22 to 51 years), CCI 2 (2 to 2) and SAPS II 51 (42 to 65), respectively. The individual characteristics of patients (numbers 1 to 14) are given in Table 1. During the observational period, 541 patients with HMs were admitted to our ICU, of whom 368 (68%) presented with respiratory failure requiring invasive mechanical ventilation. Thus, the proportion of HM patients with ARF receiving ECMO therapy was 3.8%.

**Table 1:** **Individual characteristics and outcomes**

Patient number	Malignancy	Therapy status (days since therapy)	Etiology of ARF	SAPS II	LIS	ECMO days	Bleeding	ICU and hospital outcome
1	CNS NHL	Chemotherapy (51)	Pneumonia	45	3.7	9	Minor	Died
2	Hodgkin lymphoma	Allo SCT (111)	Pneumonia	34	3.3	28^b^	Major	Died
3	ALL	Consolidation (13)	Abdominal sepsis	78	2.3	4^c^	-	Alive
4	ALL^a^	Induction on ECMO	TRALI	62	3.3	3	-	Alive
5	Burkitt lymphoma	Induction (16)	Pneumonia	63	3.8	8	-	Alive
6	ALL	Allo SCT (31)	Pneumonia	39	3.5	7	Major	Died
7	Hodgkin lymphoma	Allo SCT (33)	Pneumonia	65	3.3	18	-	Died
8	ALL	Allo SCT (203)	Pneumonia	68	3.3	10	-	Died
9	DLBCL	Induction on ECMO	Pneumonia	102	4.0	4	-	Died
10	Multiple myeloma	Auto SCT (789)	Pneumonia	43	3.7	9	Major	Alive
11	Anaplastic T-cell NHL^a^	Induction on ECMO	Pneumonia	46	3.0	25^d^	Major	Alive
12	DLBCL^a^	Induction on ECMO	NHL	36	3.3	3^c^	-	Alive
13	AML	Consolidation (34)	Pneumonia	48	3.3	34	Major	Died
14	DLBCL^a^	Induction on ECMO	NHL	56	2.3	4^d^	-	Alive

ALL, acute lymphoblastic leukemia; allo SCT, allogeneic stem cell transplantation; AML, acute myeloid leukemia; ARF, acute respiratory failure; auto SCT, autologous stem cell transplantation; CNS, central nervous system; DLBCL, diffuse large B-cell lymphoma; ECMO, extracorporeal membrane oxygenation; ICU, intensive care unit; LIS, lung injury score at ECMO baseline [30]; NHL, non-Hodgkin lymphoma; SAPS II, simplified acute physiology score at ICU admission [27]; TRALI, transfusion-related acute lung injury. ^a^Diagnosis of hematologic malignancy on ECMO; ^b^Two episodes of ECMO; ^c^ventoarterial ECMO; ^d^three episodes of ECMO.

### Hematologic malignancy

The underlying HMs were various types of aggressive non-Hodgkin lymphoma (NHL) in five patients (diffuse large B-cell lymphoma, *n* = 3; anaplastic T-cell lymphoma, *n* = 1; central nervous system NHL, *n* = 1), highly aggressive NHL in five (acute lymphoblastic leukemia, *n* = 4 and Burkitt lymphoma, *n* = 1), Hodgkin lymphoma in two, as well as acute myeloid leukemia and multiple myeloma in one patient each, respectively. The diagnosis of HM was established during ECMO support in four patients (patients 4, 11, 12, and 14). Five patients first received (immuno-) chemotherapy on ECMO, and four patients had recently received chemotherapy. Four patients had undergone ASCT within the previous year. Overall, seven patients were in the induction phase, six were in remission since 96 days (range, 39 to 292; patients 2, 3, 6 to 8, and 13), and the patient with multiple myeloma was in partial remission. All patients but the latter had curative therapeutic options regarding their underlying HMs. For further information, see Table 1.

### Etiology of acute respiratory failure

Etiology of ARF was pneumonia (*n* = 10), massive thoracic bulky disease with partial obstruction of bronchi, intrathoracic vessels, and the right heart (*n* = 2), as well as sepsis of nonpulmonary (abdominal) origin (*n* = 1), and transfusion-related acute lung injury (*n* = 1), respectively. Twelve patients fulfilled the criteria of severe ARDS. Microbiologic workup revealed pathogenic organisms in only three patients: Influenza A H1N1 (*n* = 2, patients 10 and 13) as well as histologically proven cytomegalovirus (CMV) infection (*n* = 1, patient 8). In two further patients, CMV infection was considered possible (massive replication of CMV copies in bronchoalveolar lavage fluid and plasma, patients 1 and 7). Diagnostic biopsies were performed on ECMO in five patients and revealed NHL (*n* = 2), CMV pneumonia (*n* = 1), and inconclusive results (*n* = 2). Autopsy was performed in five patients but did not reveal any further information on specific pathology or infections.

### Baseline characteristics

At baseline, the median SOFA score was 12 (11 to 13), and LIS was 3.3 (3.3 to 3.7). All patients were receiving vasopressors, and one patient underwent continuous venovenous hemofiltration. Thrombocytopenia was present in 11 patients, with a median platelet count of 35 (26 to 51) G/L, and leukocytopenia was present in five patients with a median leukocyte count of 2.1 (1.8 to 2.5) G/L.

### ECMO therapy

At the discretion of the treating intensivist, all but three patients were placed in the prone position before the start of VV ECMO. Decision for ECMO was then taken if the patient was still severely hypoxemic, or if ventilation was still unacceptably invasive.

Three patients received VA ECMO because, in addition to ARF, they presented with severe circulatory failure due to septic cardiomyopathy, leading to refractory left ventricular failure in one patient (patient 3) and life-threatening right ventricular failure in two patients, one of them with an acute subtotal occlusion of the right pulmonary artery (patient 12), and one of them with acute superior vena cava syndrome and compression of the right heart due to DLBCL masses (patient 14).

All other patients received VV ECMO because of severe ARDS only. In one patient with a recent history of ASCT, ECMO was started before invasive mechanical ventilation with the intention of preventing intubation (patient 7). However, the patient had to be intubated on the subsequent day. ECMO therapy was used for a median of 8.5 (4 to 16) days, and mechanical ventilation, for 17 (12 to 35) days.

In patients with VV ECMO, percutaneous cannulation was performed by inserting a 21- to 24-F drainage cannula into a femoral vein and a 19- to 22-F into the right jugular vein by using the ultrasound-guided Seldinger technique. In one patient (patient 7), a 31-F double-lumen cannula was placed into the right jugular vein. In all three patients with VA ECMO, venous drainage cannulas were placed into a femoral vein, whereas the blood was returned via a femoral artery in two patients and via a right-sided subclavian patch in one patient (patient 12). Cannulation in VA ECMO was performed surgically by using a semi-Seldinger cutdown technique. Sweep gas flow was set to achieve sufficient CO_2_ elimination, and the blood flow was set to achieve adequate systemic oxygenation or adequate circulatory support in VA ECMO patients.

Eleven patients received continuous intravenous infusion of unfractionated heparin to maintain an activated partial thromboplastin time of approximately 55 seconds. All but two of these patients additionally received antiplatelet therapy by intravenous prostaglandin E_1_ at doses of 2.5 to 5 ng/kg/min. Three patients received subcutaneous low-molecular-weight heparin alone.

### Interventional procedures

Both patients with circulatory failure due to massive intra-thoracic DLBCL masses were treated by interventional radiologists under emergency conditions on VA ECMO. The patient who presented with acute obstructive circulatory failure due to a highly compressed right pulmonary artery (patient 12) was treated with percutaneous implantation of a 16 × 40-mm self-expanding nitinol stent (sinus-XL; Optimed Medizinische Instrumente GmbH, Ettlingen, Germany) into the mainstem of the pulmonary artery. In the patient with severe superior vena cava (SVC) syndrome (patient 14) a 24 × 60-mm sinus-XL stent was placed into the SVC by a transjugular access. In both patients, stent placement led to an immediate restoration of blood flows and, eventually, complete recovery of the circulatory failure.

### Bleeding events and other complications

Major bleeding events were documented in five (36%) patients from these locations: upper gastrointestinal tract (*n* = 3), nasopharynx, lung, and cannula insertion site (all in one patient), and multilocular intracerebral hemorrhages (*n* = 1). Of these, only the latter patient survived and experienced full neurologic recovery after discharge. In the four nonsurvivors, bleeding was part of multiorgan failure and not the primary reason for death. One patient with septic cardiomyopathy developed severe disseminated intravascular coagulation, leading to limb ischemia, and eventually needed amputation of both lower and one upper extremity. No thrombotic events were noted in any other patient (Table 1).

### Comparison of survivors with nonsurvivors

Survivors had a significantly shorter time interval from diagnosis of the HM to the start of ECMO therapy, received less packed red blood cell units as well as single-donor platelet concentrates, and were less likely to have a history of ASCT. A trend for higher survival rates was noted in patients with shorter time intervals between ICU admission and start of ECMO therapy, shorter ECMO duration, and in patients in whom diagnosis of HM was made on ECMO. Conversely a trend for lower survival rates was noted in patients with higher LIS scores or PaCO_2_ levels at baseline, and in those with clinical signs of pneumonia (Table 2).

**Table 2:** **Cohort characteristics and outcomes**

	All patients ***N*** = 14	Survivors ***n*** = 7	Nonsurvivors ***n*** = 7	***P*** value
**Characteristics at ICU admission**				
Age	32 (22–51)	23 (21–44)	48 (30–58)	0.14
Male sex	8	4	4	1.00
CCI	2 (2–2)	2 (2–2)	2 (2–4)	0.48
SAPS II	51 (42–65)	56 (43–63)	45 (39–68)	0.90
Prior allogeneic stem cell transplantation	4	0	4	<0.01
Days from diagnosis of HM to ECMO	87 (6–907)	0 ((−1)–116)	759 (69–1,228)	0.04
Days from ICU admission to ECMO	2 (0–3)	1 (0–2)	3 (1–11)	0.08
Days from intubation to ECMO	2 (1–5)	1 (0–2)	3 (1–9)	0.17
**Characteristics at ECMO baseline**				
SOFA score	12 (11–13)	12 (12–19)	12 (11–15)	0.79
Lung injury score	3.3 (3.3–3.7)	3.3 (2.3–3.7)	3.5 (3.3–3.8)	0.09
PaO_2_/FiO_2_ ratio	60 (53–65)	63 (51–106)	60 (48–61)	0.44
pH	7.29 (7.23–7.37)	7.29 (7.23–7.39)	7.37 (7.21–7.46)	0.52
PaCO_2_, mm Hg	49 (43–59)	41 (38–49)	55 (48–72)	0.05
Lactate, m*M*	2.2 (1.6–4.8)	3.8 (1.6–7.1)	2.0 (1.0–5.0)	0.34
Hemoglobin, g/dl	9.4 (8.8–10.4)	9.4 (8.6–10.5)	9.8 (8.4–10.4)	0.90
Leukocytes, G/L	6.0 (2.5–12.6)	11.0 (3.6–17.0)	5.6 (2.0–12.8)	0.34
Platelets, G/L	38 (30–113)	64 (29–201)	35 (10–88)	0.21
Prothrombin time, %	70 (43–74)	50 (18–72)	73 (64–78)	0.22
Fibrinogen, mg/dl	436 (220–522)	473 (160–627)	421 (294–531)	1.00
Etiology of ARF determined^a^	7	5	2	0.29
Clinical diagnosis of pneumonia	10	3	7	0.07
**Characteristics during ECMO**				
Venoarterial ECMO	3	3	0	0.19
Duration of ECMO therapy, days	8.5 (4–16)	4 (3–9)	10 (7–28)	0.08
Diagnosis of HM on ECMO	4	4	0	0.07
Chemotherapy on ECMO	5	4	1	0.27
Vasopressors	14	7	7	na
Hemofiltration	5	2	3	1.00
Major bleeding events	5	1	4	0.27
Number of packed red blood cell units	8 (4–14)	4 (3–8)	14 (6–27)	0.02
Number of platelet concentrates	5 (1–17)	2 (0–5)	23 (14–26)	0.01
**Outcome**				
ICU LOS, days	22 (14–42)	22 (21–77)	18 (11–40)	0.12
Hospital LOS, days	56 (44–101)	63 (49–110)	45 (15–133)	0.46
ICU and hospital survival, *n* (%)	7 (50%)			

Data are given as median and interquartile range or *n*, respectively; ARF, acute respiratory failure; CCI, Charlson comorbidity index [26]; HM, hematologic malignancy; ICU, intensive care unit; ECMO, extracorporeal membrane oxygenation; LOS, length of stay; na, not applicable; SAPS II, Simplified Acute Physiology Score at ICU admission [27]; SOFA score, Sequential Organ Failure Assessment Score at ECMO Baseline [29]; ^a^microbiologic pathogen detected *or* histologic proof of HM in lung biopsy.

### Survival and hematologic outcome

Survival off ECMO episodes was 59% (10/17). One of the two patients with multiple episodes of ECMO survived and was discharged after the third application of ECMO, whereas the other patient died during the second ECMO episode. All patients with a history of ASCT died. Seven (50%) patients survived the ICU and hospital stay. Their long-term survival was 100% after a median follow-up of 36 (10 to 58) months. At the time of the last visit, five survivors were in complete remission of their HM, whereas the patient with multiple myeloma remained in partial remission, and one patient with anaplastic T-cell lymphoma had experienced relapse. All but one patient in need of further antineoplastic treatment received the regularly scheduled number of chemotherapy courses without dose reduction.

## Discussion

Recent data show impressive improvements in the outcomes of critically ill patients with HMs [1]. However, mortality rates still remain at >50%, if invasive mechanical ventilation becomes necessary because of ARF [1, 2, 4, 5, 9]. Thus, novel strategies to treat these patients are desperately needed. This is the first report on the use of ECMO in a series of patients with HMs and ARF. We observed a remarkable short- and long-term survival of 50%, despite the presence of numerous factors known to be associated with adverse outcome [13]: First, all patients presented with at least three organ failures. Second, intensive diagnostic efforts, including lung biopsy, failed to identify causative pathogens in almost all patients with pneumonia. Third, four (29%) patients had undergone ASCT within the previous year. The finding that all four patients with a history of ASCT died, underlines the negative prognostic impact of allogeneic stem cell transplantation in critical illness. However, two of our patients may not have been good candidates for aggressive ICU management because of active graft-versus-host disease and thrombotic microangiopathy, which is known to be associated with poor outcome [31, 32]. All of the 10 remaining patients fulfilled the criteria for a full-code ICU management, as recently proposed by an international expert consensus on critically ill cancer patients [13]. Their long-term survival was 70% even in the presence of multiorgan failure, severe and persistent thrombocytopenia, and/or leukocytopenia, multiple episodes of ECMO, the administration of chemotherapy, or other complex interventions before or during ECMO. Moreover, all but one surviving patient had favorable hematologic outcomes during the follow-up period.

The high rate of severe bleeding events (five (36%) of 14) raises concerns about the safety of ECMO in patients with persistent thrombocytopenia. Importantly, four of five bleeding events occurred in the context of terminal multiorgan failure and were not likely to be the primary cause of death. However, according to our experience, keeping platelet count above the recommended threshold of 80 G/L [25] does not seem to be feasible in most patients with HMs. Thus, avoiding any additional risk factors for bleeding seems of major interest. We refrained from using any anticoagulation therapy for a total of 14 days in one persistently thrombocytopenic patient by using a heparin-coated system (HLS Set Advanced 7.0, Maquet Cardiopulmonary AG, Rastatt, Germany). The gas exchange remained excellent during the whole period, and no signs of hemolysis or coagulation activation were noted. To minimize the risk of bleeding in patients refractory to platelet transfusions, we suggest considering a “low or no” strategy concerning anticoagulation.

This study has several limitations. First, the retrospective design of the investigation does not allow conclusions concerning the efficacy of ECMO in these patients. Second, the number of patients is small. Third, our data reflect the experience of a single institution specifically devoted to the treatment of critically ill cancer patients and patients with ARDS, including extracorporeal lung support. Furthermore, it must be acknowledged that our cohort of patients represents only a very small proportion of critically ill HM patients with respiratory failure. Thus, our series is highly selective, and our findings may not be generalized.

Despite the promising results of our report, applying ECMO to patients with HMs cannot be regarded as an established therapy. Its efficacy and associated risks still remain to be established. Further research should focus on following questions: Which patients are likely to benefit from ECMO? When and under which circumstances should ECMO be initiated? What are the possible risks apart from complications associated with cannulation, infections, and bleeding events? Which measures must be taken to make ECMO as safe as possible in these vulnerable patients? Can ECMO be safely performed in spontaneously breathing patients with HMs and ARF to prevent endotracheal intubation?

Until these issues are settled, we propose to apply ECMO to patients with HMs exclusively under the following circumstances: (a) patients qualify for a full-code ICU management according to current recommendations on critically ill cancer patients [13], (b) lung-protective ventilation strategies, including supportive measures like prone positioning, result in inadequate gas exchange [24, 33], (c) patients are included into yet to establish prospective research protocols, (d) a close cooperation of intensivists, hematologists, vascular surgeons, and perfusionists (if part of the local standard) is warranted, (e) general guidelines for the management of patients on ECMO are available and being followed [25], and (e) issues concerning anticoagulation and platelet transfusion are considered with special respect.

## Conclusions

In conclusion, performing ECMO in carefully selected patients with HMs and severe ARF can be associated with favorable short- and long-term survival. This finding may even apply to patients with multiorgan failure, severe cytopenia, newly diagnosed HMs, as well as to those who need chemotherapy or complex interventional procedures on ECMO.

## Key messages

- ECMO is feasible in patients with hematologic malignancies and acute respiratory failure.
- These patients are at increased risk for severe bleeding events.
- Short- and long-term survival may be significant in selected patients.

## Authors’ information

Peter Schellongowski; on behalf of the Arbeitsgruppe für hämato-onkologische Intensivmedizin der Österreichischen Gesellschaft für Internistische und Allgemeine Intensivmedizin und Notfallmedizin (ÖGIAIN).

## Abbreviations

ARDS: Acute respiratory distress syndrome
ARF: acute respiratory failure
ASCT: allogeneic stem cell transplantation
CCI: Charlson comorbidity index
DLBCL: diffuse large B-cell lymphoma
ECMO: extracorporeal membrane oxygenation
HM: hematologic malignancy
ICU: intensive care unit
LIS: lung injury score
NHL: non-Hodgkin lymphoma
SAPS II: Simplified Acute Physiology Score II
SOFA score: Sequential Organ Failure Assessment score
SVC: superior vena cava
VA: venoarterial
VV: venovenous.
